# Simple and Green Process for Silk Fibroin Production by Water Degumming

**DOI:** 10.1021/acsomega.4c05531

**Published:** 2025-01-03

**Authors:** Ipek Atay, Ecenaz Asad, M. Baris Yagci, Saliha Sürme, Ibrahim Halil Kavakli, Emel Yilgör, Iskender Yilgör

**Affiliations:** † Chemistry Department, Koc University, Sariyer, Istanbul 34450, Turkey; ‡ Koc University Surface Science and Technology Center (KUYTAM), Koc University, Sariyer, Istanbul 34450, Turkey; § Molecular Biology and Genetics Department, Koc University, Sariyer, Istanbul 34450, Turkey; ∥ Chemical and Biological Engineering Department, Koc University, Sariyer, Istanbul 34450, Turkey

## Abstract

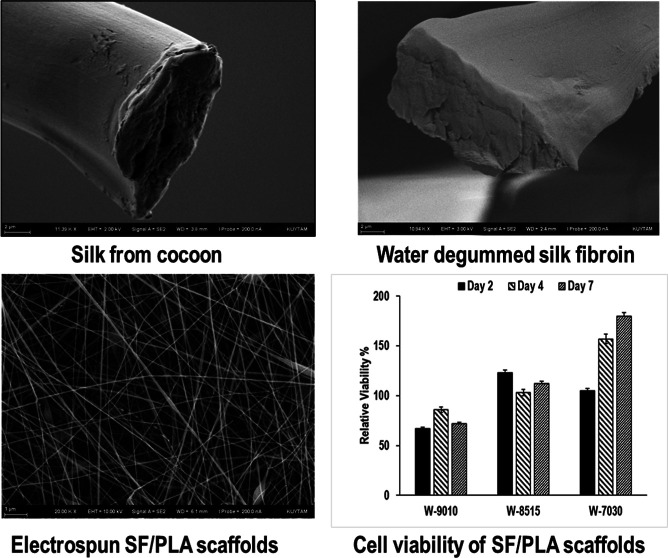

Silk fibroin (SF), a natural polymer with very desirable physicochemical and biological properties, is an ideal material for crafting biocompatible scaffolds in tissue engineering. However, conventional methods for removing the sericin layer and dissolving SF often involve environmentally harmful reagents and processes, requiring extensive dialysis procedures to purify the fibers produced. Such processes may also damage the surface and bulk properties of the SF produced. Here, we report a simple, green water degumming method, in which almost complete sericin removal of 30% by weight is achieved in 6 h in boiling water. The SF produced is easily dissolved in formic acid/orthophosphoric acid (90/10, 85/15, and 70/30) mixtures, eliminating the need for salts like LiBr and CaCl_2_ followed by dialysis and freeze-drying, thus simplifying the process significantly. Additionally, our findings demonstrate significantly enhanced cell viability in electrospun poly(lactic acid)/SF blends. Overall, SF production via water degumming offers an eco-friendly pathway for generating bioactive scaffolds in tissue engineering applications.

## Introduction

Silk fibroin (SF), a protein extracted from the silk fibers produced in the silk glands of spiders, mites, and *Bombyx mori* silk moths, has been used as textile fibers, sutures, and wound dressings since very early ages.^1^ Since 1990s, with the rise of tissue engineering as an interdisciplinary field to provide a solution to the need to repair or replace dysfunctional tissues or organs, SF has been studied as a scaffolding material.^1,2^ Gly-Ser-Ala amino acid sequence in silk backbone, resembling cellular extracellular matrix proteins,^1^ along with its mechanical stability and resistance to biodegradation, has contributed to SF’s popularity as a biomaterial,^1,3^ particularly in terms of biocompatibility,^4^ angiogenesis,^5^ blood compatibility,^6^ and antithrombogenicity.^7^ SF has been extensively studied for various tissue engineering applications, including corneal,^8^ bone,^9^ skin,^10^ dermal,^11^ and biosensor^12^ applications.

Silk is a water-insoluble protein comprising two components, namely, SF and a gum-like sericin protein that keeps SF filaments together.^1^ Based on the reports of silk sericin causing allergic and immunogenic reactions in biomedical applications,^13^ removal of the sericin protein, which constitutes 26.5–33 wt % of the silk,^14^ by a process called degumming has become the standard processing method to obtain SF.^1^ Degumming relies on the solubility of the sericin protein in aqueous solutions under the appropriate conditions. Therefore, various degumming procedures, mostly under mildly basic or acidic conditions, in aqueous solutions with enzymatic reagents, in a chemical-free environment, or others have been developed.^15−26^ The critical aim of these processes is to completely remove sericin without any damage to the surface and/or bulk properties of SF fibers produced, which include molecular weight, mechanical integrity, and surface properties of the filaments.^15−26^

Degumming by boiling silk cocoons in alkali solutions containing sodium bicarbonate (NaHCO_3_), sodium carbonate (Na_2_CO_3_), or ammonia (NH_3_) has become the most common method due to fast sericin removal.^15,17^ Processes involving alkaline reagents rely on the interaction of anions and cations generated with the amide groups in the sericin protein, which increases its solubility in water. This results in the facile cleavage of the sericin layer off the SF filaments into the aqueous degumming solution.^17^ However, factors such as possible damage to the SF filaments and molecular weight reduction due to hydrolysis have driven the focus to alternative techniques such as using urea or surfactants/soaps as degumming reagents.^17^ Although using urea or surfactants results in less damage to the fibers and produces SF with improved elasticity, these processes are not time-efficient.^17^ Although degumming in boiling water has been reported, it has not been optimized for complete removal of sericin to produce pure SF.^16,18^ Interestingly, various studies have shown sericin to be a biocompatible and even bioactive protein on its own.^27^ The effect of the residual sericin on the performance of SF as a biomaterial, especially on cell viability, was investigated and reported by different groups.^28,29^

In order to be fabricated into the desired scaffold form such as a film, sponge, fiber, or hydrogel, after the sericin removal, the degummed SF has to be dissolved in a solvent system by breaking the hydrogen bonds and hydrophobic interactions within its structure.^14^ Dissolution of silk fibers in weakly acidic solutions goes back to 1920s, where formic acid (FA) (HCOOH) (p*K*_a_ = 3.75) and orthophosphoric acid (OPA) (H_3_PO_4_) (p*K*_a1_ = 2.12) with salts such as zinc chloride (ZnCl_2_) were used as solvents.^30^ Interestingly, degumming using Na_2_CO_3_ followed by dissolution in lithium bromide (LiBr) became popular in 1930s, which is still used as the standard process.^30^ Dissolution of SF using inorganic acids, ionic liquids, or salts containing organic/aqueous solvent systems such as mixtures consisting of CaCl_2_/ethanol/water,^31^ CaCl_2_/FA,^32^ and calcium nitrate Ca(NO_3_)_2_/ethanol/water mixtures^14,33^ has also been explored. CaCl_2_-containing solvent systems have become very popular due to the low cost and fast dissolution of silk fibers. However, removal of the salt residues requires very long, extensive dialysis processes,^14^ and even at the end of the dialysis process, various studies have found Ca^2+^ residues within the regenerated SF, which could affect its biocompatibility and other properties.^34^ OPA has been reintroduced and studied as a solvent for the dissolution of SF starting from 1980s.^35^ Degummed silk fibers were dissolved in OPA/FA mixtures to facilitate wet spinning of silk.^36^ In other studies, silk fibers were dissolved in cold aqueous OPA to minimize possible depolymerization of SF by phosphoric acid, even at room temperature.^36,37^

In this study, we explored water degumming of SF from *B. mori* cocoons. For comparison, aqueous alkali (Na_2_CO_3_) degummed SF was also produced. Then, we investigated dissolution of SF fibers in salt-free FA/OPA solutions for the first time, where, by the addition of small amounts of OPA into FA, fast dissolution of SF fibers at ambient conditions was achieved, as compared to pure FA.^38−45^ In the water degumming process, a maximum sericin removal of 30% by weight was achieved when the weight ratio of silk cocoons to water was in 0.3–0.6 wt %. Complete removal of sericin was confirmed by scanning electron microscopy (SEM) images taken on an hourly basis and gravimetric measurements during the degumming process. After dissolution in FA/OPA mixtures, SF was treated with ethanol to promote β-sheet formation to improve degradation stability. Ethanol-treated SF solutions were solvent-cast as a film as well as electrospun by blending with poly(lactic acid) (PLA). X-ray diffraction (XRD) diffractograms were obtained to evaluate the effect of water degumming and OPA dissolution on β-sheet formation. MTT cell viability assays were applied on the electrospun SF scaffolds with the HepG2 hepatocarcinoma cell line over 6 days, with measurements taken on days 2 and 6. Interestingly, the highest cell viabilities were obtained on water-degummed SF scaffolds. Our results show that boiling water degumming followed by FA/OPA dissolution can be used to obtain highly stable, biocompatible, and bioactive SF.

## Materials and Methods

### Materials

Silk cocoons from *B. mori* were kindly provided by IDEALAB Koç University. FA (98%), OPA (98%), ethanol, dimethyl sulfoxide (DMSO), and sodium carbonate (Na_2_CO_3_) were obtained from Merck and were used as received. Deionized and triple-distilled water at pH 5.0 was produced in our laboratory. PLA filaments were obtained from 3DFab and used as received. For the cell viability assay, the HepG2 hepatocarcinoma cell line was obtained from ATCC. The PanReac Applichem Biochemica MTT Reagent was used as received.

## Experimental Methods

### Water Degumming of Silk Cocoons

SF was regenerated by removing the sericin layer in boiling water (W) and in a dilute aqueous alkali solution (A). In water, degumming, cut, and cleaned cocoons were boiled in distilled water at pH 5. The weight ratio of the silk cocoons to water was 0.6% w/v and 0.3% w/v. The cocoons were boiled for 6 h at 95–97 °C, and samples were taken at each hour during extraction to quantitatively determine the amount of sericin removal. Degummed fiber samples were completely dried in a vacuum oven and weighed to determine the amount of sericin removal. Experiments were performed in triplicate.

For alkaline degumming (A), 2.65 g (0.066 mol) of Na_2_CO_3_ was dissolved in 500 mL of distilled water to obtain a 0.13 M aqueous solution with a pH of 11. The cut and cleaned cocoons were boiled for 30 min at 95–97 °C. The degummed fibers were washed repeatedly with distilled water until neutral and vacuum-dried until constant weight.

### Dissolution of Degummed SF in FA/OPA Mixtures

The degummed SF obtained by water (W) and alkaline (A) processes was dissolved in FA/OPA mixtures, with 90/10, 85/15, and 70/30 v/v ratios at 23 °C in 3 h to produce solutions with an SF concentration of 6% by weight. By keeping FA in excess, a possible depolymerization effect of OPA on SF, even at room temperature, was minimized. The samples were coded by the degumming method, followed by the volumetric ratio of FA to OPA, as W-9010, W-8515, W-7030, A-9010, A-8515, and A-7030. Water degumming technique in conjunction with dissolution of SF in salt-free FA/OPA mixtures is reported for the first time in this study.

### Treatment of SF Solutions with Ethanol

SF solutions in FA/OPA mixtures were coagulated in ethanol at 23 °C to induce random coil to β-sheet transformation and improve biodegradation resistance of SF. Coagulation was obtained by dropwise addition of 20 mL of 6% by weight SF solutions in FA/OPA into 300 mL of ethanol under constant stirring. Coagulated SF was recovered by filtration and dried overnight at 23 °C and then in a 60 °C air oven until constant weight.

### Electrospinning of SF/PLA Blends to Obtain Biocomposite Webs

It is fairly difficult to electrospin pure SF solutions; therefore, PLA was used to improve the electrospinnability of SF. To prepare blend solutions for electrospinning, ethanol-treated SF pellets (0.71 g) were dissolved in 4.0 g of FA at 23 °C to obtain 15 wt % solutions. 3 g of PLA pellets was dissolved in 50 mL of 70/30 v/v mixture of chloroform/acetone at 23 °C to obtain a 6 wt % PLA solution. 3 g of SF solution was added into 1 g of PLA solution under stirring at room temperature to obtain an SF/PLA ratio of 7.5/1 in each solution. Solutions were stirred for 2 h and electrospun immediately to avoid precipitation. SF/PLA blend solutions were electrospun using a 10 mL syringe, with a 9 cm distance from the needle tip to the collector, 21 kV and 0.1 mL/h flow rate. Electrospun samples were dried at 23 °C until constant weight.

### Characterization Methods

SEM images were obtained on a Zeiss Ultra Plus Field Emission scanning electron microscope. To prevent surface overcharging, electrospun SF samples were coated with a 10 nm layer of gold prior to imaging. XRD measurements were taken on the SF flakes, on a Bruker XRD diffractometer with CuKα source at 5–60 2θ°, with a speed of 2° per minute. Static water contact angle measurements were performed on a Krüss G-10 goniometer, using a 5 μL droplet at 4 points on each film sample. Films were prepared by casting SF/PLA solutions prepared for electrospinning into Teflon molds and drying until constant weight under ambient conditions. Surface chemical compositions of ethanol treated and extracted SF samples were determined by X-ray photoelectron spectroscopy (XPS) on a Thermo Scientific K-Alpha XPS equipped with a monochromatic Al Kα excitation source (1486.6 eV) and a hemispherical analyzer. Dry samples were exposed to 400 μm X-ray spot size, and 50.0 eV pass energy was set to increase spectral resolution. The takeoff angle was set to 90°. A flood gun was employed to reduce surface charging, and all spectra were referenced to the C 1s primary signal at 284.5 eV. Atomic compositions were determined by using the supplied Avantage 5.9 software. Attenuated total reflection–Fourier transform infrared (ATR–FTIR) spectra were collected on a Thermo Scientific Nicolet iS20 spectrometer on fiber samples with a resolution of 4 cm^–1^. Intrinsic viscosities were determined using Ubbelohde viscometers in FA at room temperature.

### In Vitro Cell Viability Assay

Cell viability assays were performed by using the colorimetric (4,5-dimethyl-2-thiazolyl)-2,5-diphenyl tetrazolium bromide MTT assay with the HepG2 hepatocarcinoma cell line on the SF/PLA-electrospun biocomposite samples. The samples underwent sterilization under a 254 nm UV light for 30 min on both sides. Subsequently, sterilized samples were placed in a 96-well plate and seeded with 3 × 10^5^ cells per 2 mL of complete DMEM culture media in each well and incubated for 48 h at 37 °C under 5% CO_2_. Afterward, 2 mL of 5 mg/mL MTT reagent in PBS was applied on the seeded samples and allowed to incubate for 4 h at 37 °C. Then, the DMEM and MTT mixture was discarded, and the resulting metabolic formazan salt was dissolved in DMSO/ethanol mixture and transferred to a new 96-well plate. Optical density measurements were performed in each well by taking the 600 nm absorbance readings of the solutions on days 2, 4, and 7 using a Synergy H1Microplate Reader (Bio-Tek Instruments, Winooski, VT, USA). The same procedure was applied on an empty cell culture plate, and the readings obtained in the same manner were taken as reference as 100% cell viability. Relative cell viability was determined as the ratio of the optical density of the sample to the optical density of the reference plate.

## Results and Discussion

**Figure 1:**
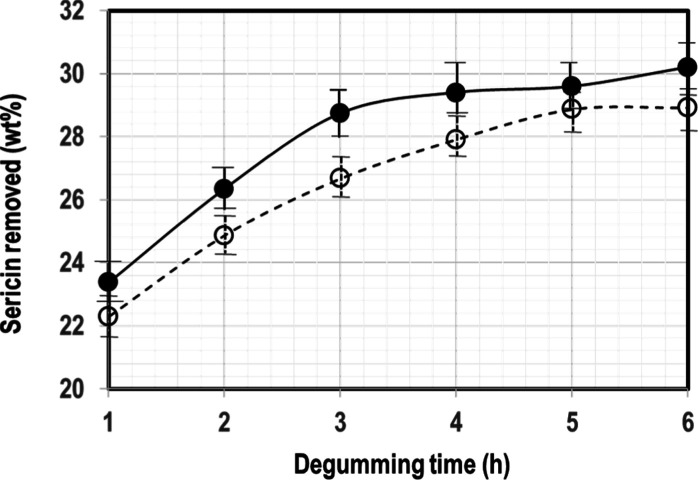
Gravimetric determination of sericin removal by degumming in boiling water as a function of time and cocoon content. (●) 0.3% w/v and (o) 0.6% w/v.

The main aim of this study was water degumming of SF from *B. mori* cocoons. To demonstrate the efficiency of water degumming, conventional alkali degumming was also performed. Figure 1 gives the effect of the water/cocoon ratio on hourly sericin removal by water degumming. The maximum amount of sericin removed was 30% and 29% by weight in 6 h for cocoon/water ratios of 0.3% w/v and 0.6% w/v, respectively. These values are in line with the reported 26.5–33 wt % sericin content in the silk,^14^ clearly indicating the effectiveness of the “green” water degumming process.

### Morphology of the Degummed Silk Fibers

**Figure 2:**
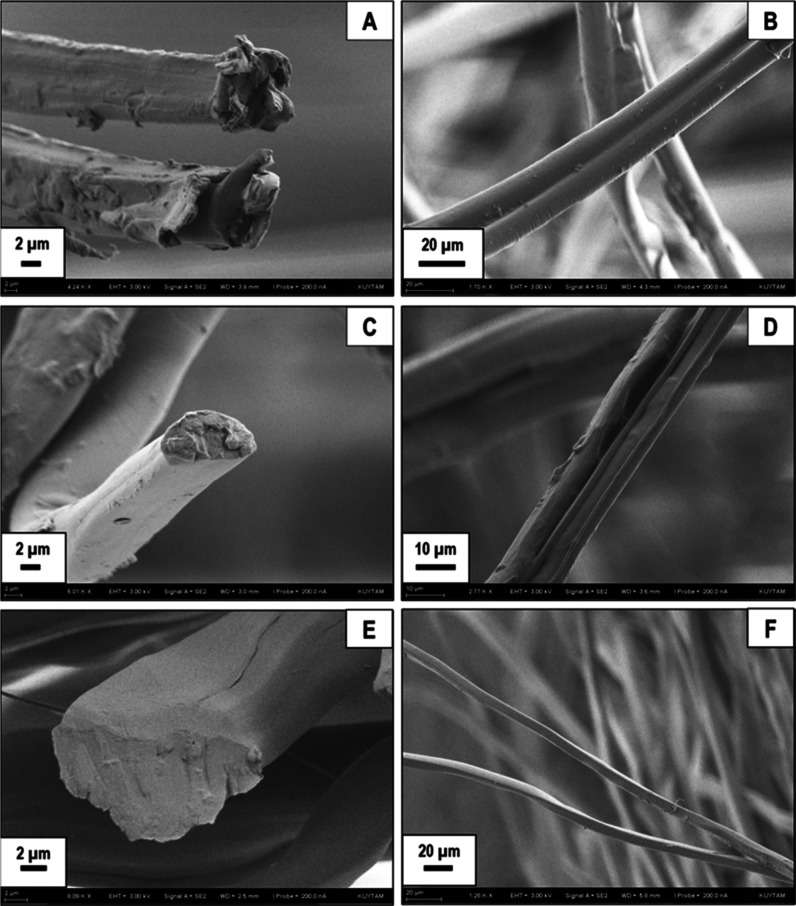
SEM images of pristine silk fibers (A,B) obtained from silk cocoons, silk fibers obtained after 3 h of degumming (C,D), and after 6 h of degumming (E,F) in boiling water, where all sericin is removed.

The effectivity of boiling water degumming method on sericin removal from silk fibers was examined by SEM studies. SEM measurements were performed on pristine and water-degummed silk fibers. To understand the influence of degumming time, samples were removed from the degumming medium after 1, 3, and 6 h of boiling and dried under vacuum at 23 °C until constant weight. The fibers were then cut to obtain cross-sectional surfaces, which were coated with a 10 nm-thick gold layer prior to imaging. SEM images are provided in Figure 2.

Figure 2A,B clearly shows a fairly thick sericin layer surrounding the pristine SF filaments. After 3 h of water boiling, an individual fibroin strand is observed to be free of the outer sericin layer, as shown in Figure 2C, while Figure 2D shows two adjacent SF filaments still held together by a partially cleaved sericin layer. After 6 h of water degumming, sericin-free SF filaments can clearly be seen in Figure 2E,F. As expected, these results demonstrate that sericin degumming in boiling water is time-dependent. The SEM images provided show that break down and removal of the outermost sericin layer occurs within the first 3 h of boiling. Complete removal of the sericin layer is achieved in about 6 h of degumming.

**Figure 3:**
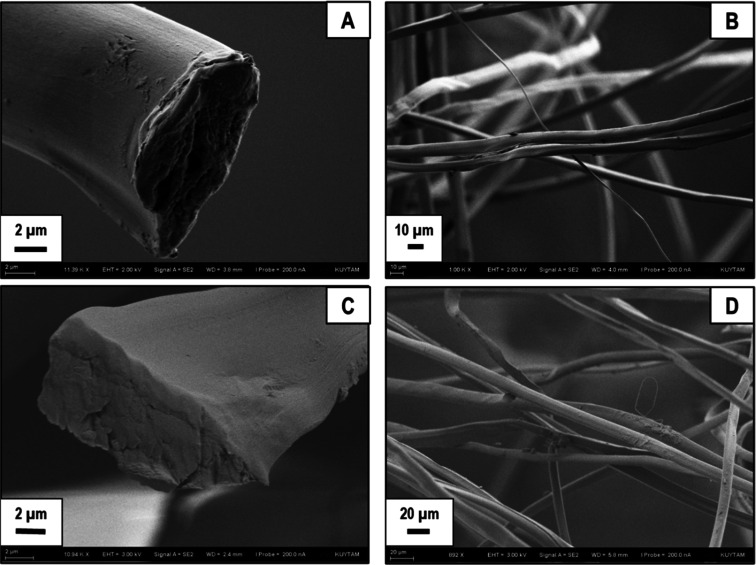
SEM images of silk fibers obtained by degumming in boiling water for 6 h (A,B) and in an aqueous alkaline solution for 30 min (C,D).

Comparative SEM images of sericin-free silk fibers degummed by boiling water for 6 h (Figure 3A,B) and boiling aqueous alkaline solution for 30 min (Figure 3C,D) are reproduced in Figure 3 for comparison. Sericin-free SF filaments can be seen in all of the SEM images provided. These results demonstrate that water boiling is a simple, effective, and green method for sericin removal, yielding similar results to alkaline degumming, which requires a tedious and time-consuming salt removal process after degumming.^46^ Furthermore, intrinsic viscosity measurements conducted in FA at room temperature did not indicate major differences in the molecular weights of water or alkaline degummed or SF, which were determined to be 0.29 ± 0.02 and 0.27 ± 0.02 dL/g, respectively.

### XPS Studies

**Figure 4:**
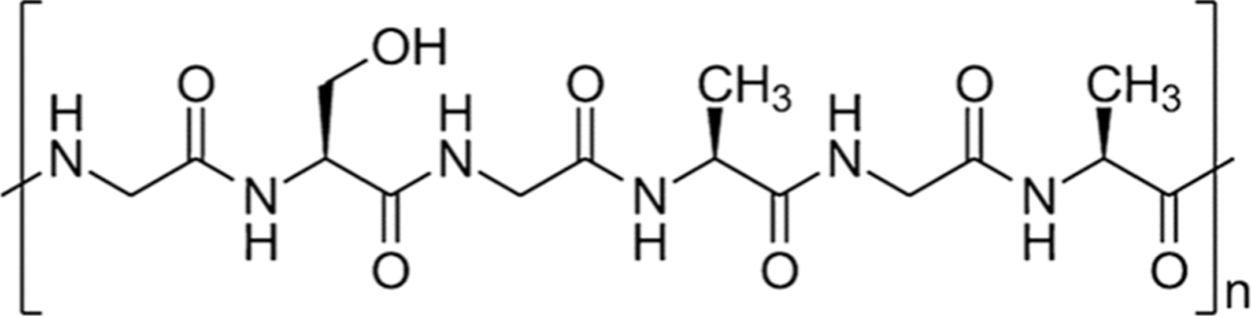
Chemical structure of the repeat unit of SF protein.

Repeat unit of SF protein is provided in Figure 4.^47^ XPS is a powerful technique for elemental analysis of polymeric materials. Since in this study different degumming techniques and dissolution methods were employed, we utilized XPS to determine the amount of residual phosphate in the SF samples originating from the dissolution process.

**Figure 5:**
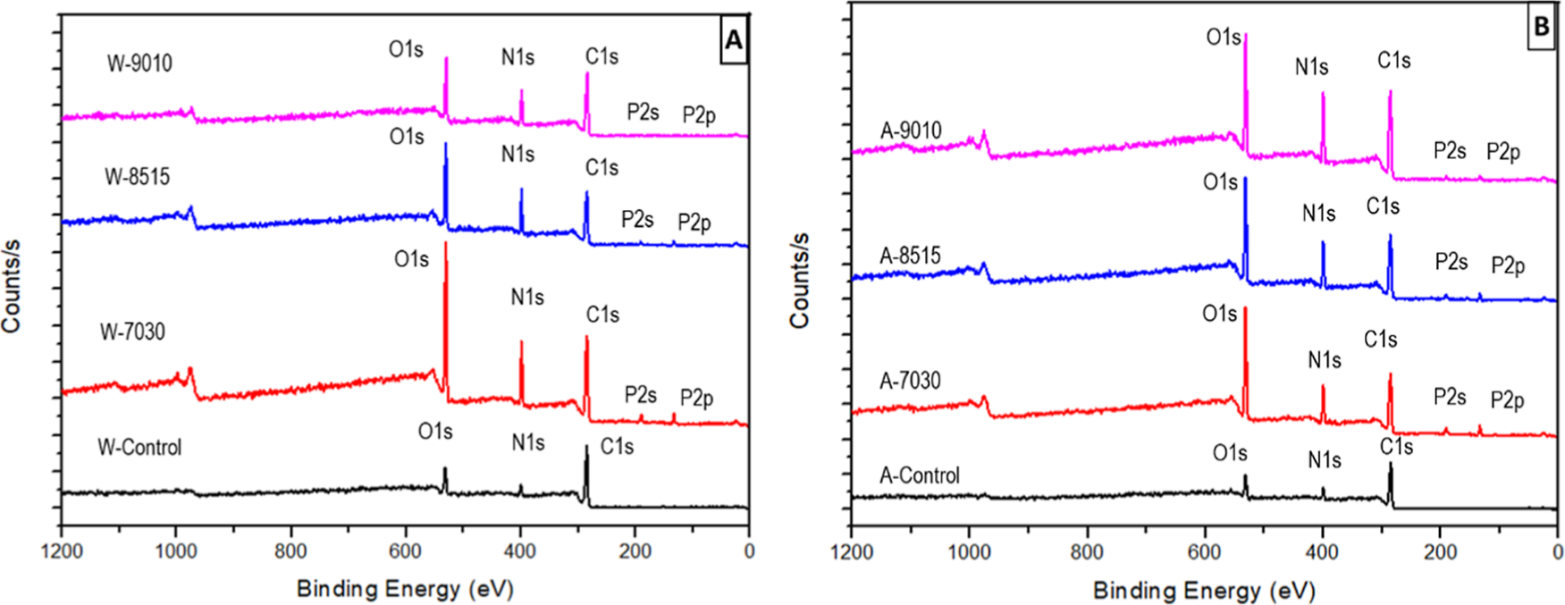
XPS survey scan of (A) water-degummed and (B) alkali-degummed SF samples, dissolved in FA/OPA with different compositions and coagulated in ethanol.

XPS survey scans of SF samples showing the presence of carbon (C), nitrogen (N), oxygen (O), and very small amounts of phosphorus (P) on their surfaces are provided for water-degummed samples (Figure 5A) and alkali-degummed samples (Figure 5B). W-Control and A-Control indicate degummed samples without any FA/OPA dissolution. Survey scans given in Figure 5 show C 1s (BE = 284.5 eV), N 1s (BE = 400.6 eV), and O 1s (BE = 532.0 eV) on all surfaces together with very small amounts of P (P 2s BE = 187.2 eV and P 2p BE = 133.5 eV), except in the control samples, indicating incomplete removal of the phosphoric acid residues by vacuum drying at 60 °C.

**Figure 6:**
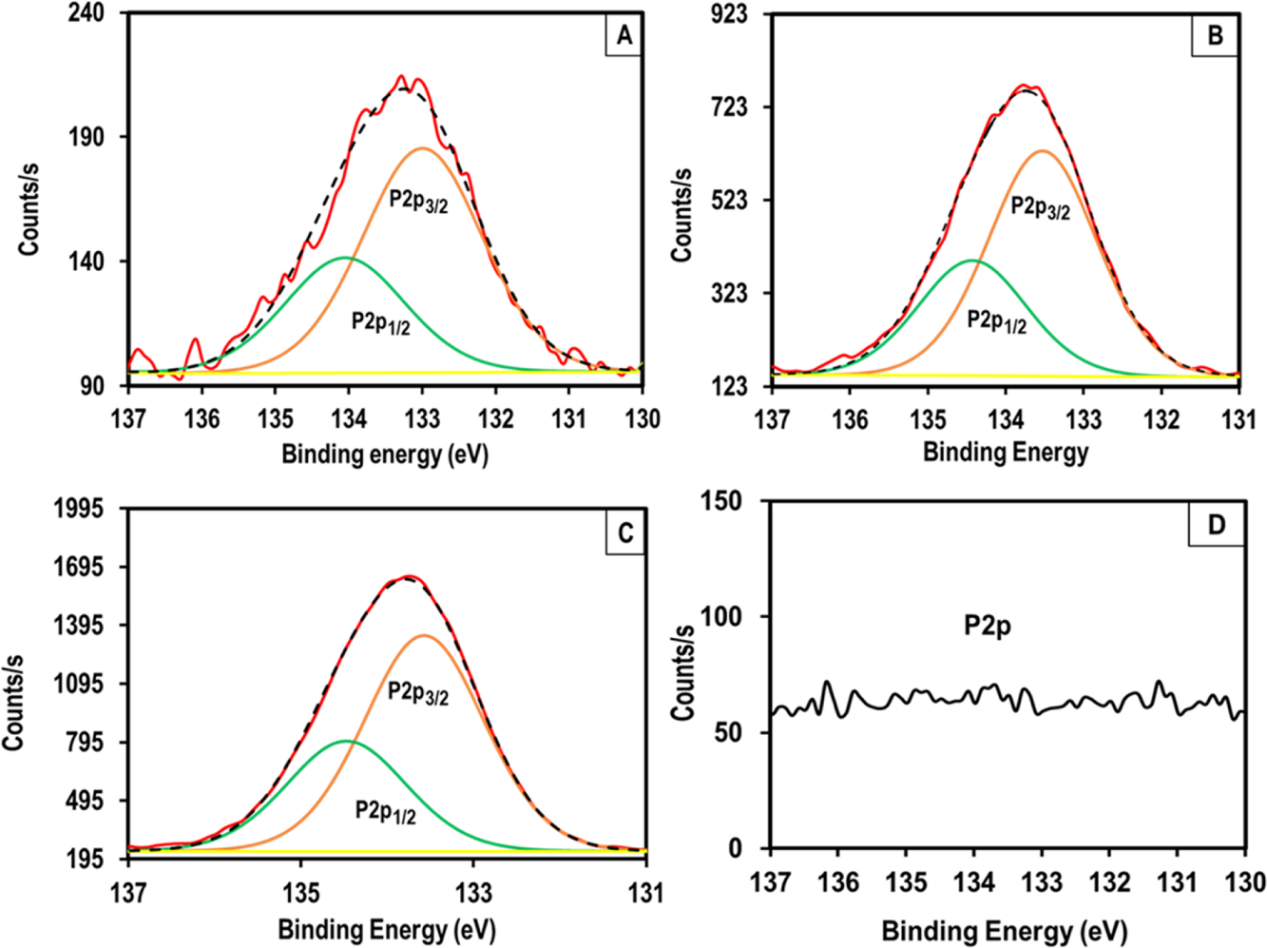
Expanded and deconvoluted XPS peaks for P 2p (BE = 133.5 eV) scans for water-degummed samples dissolved in FA/OPA mixtures with different compositions and coagulated in ethanol. (A) W-9010, (B) W-8515, (C) W-7030, and (D) control sample.

Expanded and deconvoluted XPS peaks for P 2p (BE = 133.5 eV) scans for water-degummed samples dissolved in FA/OPA mixtures with different compositions and coagulated in ethanol are provided in Figure 6, together with the control sample, which was water degummed but not dissolved in the FA/OPA mixture. As expected, the control sample (Figure 6D) does not show any P 2p peak. On the other hand, as can clearly be seen in Figure 6A–C, as the amount of OPA in the FA/OPA mixture increases, counts/s values also increase, indicating higher amounts of residual phosphorus in these samples. Very similar results were also obtained for alkali-degummed samples.

**Figure 7:**
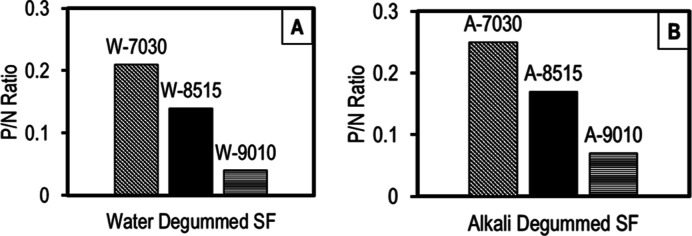
Atomic P/N ratios determined from XPS scans for (A) water- and (B) alkali-degummed SF samples dissolved in FA/OPA mixtures with different compositions.

For a better comparison, atomic P/N ratios determined from XPS scans are provided in Figure 7 for water (W)- and alkali (A)-degummed samples. As can be seen here, a significant decrease in the P/N ratio is observed with a decrease in the amount of OPA in the FA/OPA solvent mixture. Since phosphates play vital roles in various cell functions, such as in intra- and extracellular protein regulation, pH balance and signaling, and introduction of small amounts of phosphates into SF through the FA/OPA dissolution process, they may contribute favorably to the cell viability of the SF scaffolds.^48^

### XRD Studies

**Figure 8:**
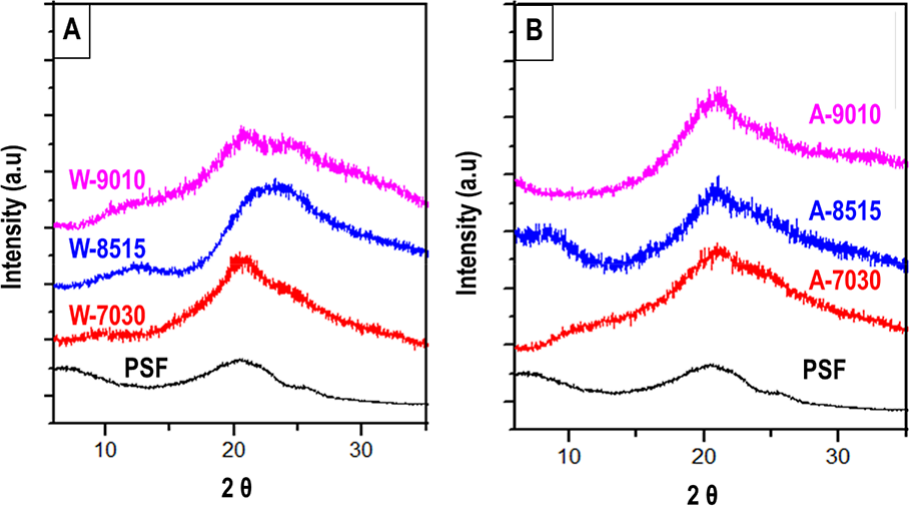
XRD patterns of PSF and SF films produced after (A) water degumming and (B) alkaline degumming. All films were cast from FA/OPA solutions with different compositions using SF flakes coagulated in ethanol.

SF protein consists of crystalline and amorphous regions where hydrophobic crystalline regions are dispersed within an amorphous hydrophilic matrix. Amorphous regions consist of random coil and α-helix, whereas crystalline regions have β-sheet structures.^38,49^ Transformation of α-helix and random coils into ordered β-sheet structures during processing gives SF unique properties such as improved mechanical properties and resistance to biodegradation.^50^ Amorphous to crystalline transformations in SF are dependent on the degumming method and conditions, solvent system used in dissolution, and post-treatment methods (such as alcohol treatment).^50^ In this study, the effect of water and alkaline degumming and the role of FA/OPA solvent composition on the morphology and crystallinity of SF was examined by XRD studies. Figure 8 gives X-ray diffractograms of pristine SF as a reference (SF) and water- or alkali-degummed SF dissolved in FA/OPA solutions with different compositions coagulated in ethanol.

A pristine silk film shows 2 weak β-sheet peaks at 2θ 20.5 and 25.5°. Degumming is expected to result in a decrease in β-sheet formation in regenerated SF.^31^ However, as seen in Figure 7, the degumming method used or dissolution in FA/OPA did not result in a significant decrease in the β-sheet structure. All samples show well-defined β-sheet peaks around 2θ 20° and weak β-sheet peaks around 2θ 24°. Dissolution in 70/30 and 90/10 FA/OPA solutions results in similar sharp β-sheet peaks around 20° and very broad, weak, shoulder-like peaks around 24° in both degumming methods corresponding to *d*-space values of 0.43 and 0.37 nm, respectively. These results suggest the formation of a silk II β-sheet structure and partial recovery of β-sheet structures during the SF regeneration process.^50^

### FTIR Spectroscopy

**Figure 9:**
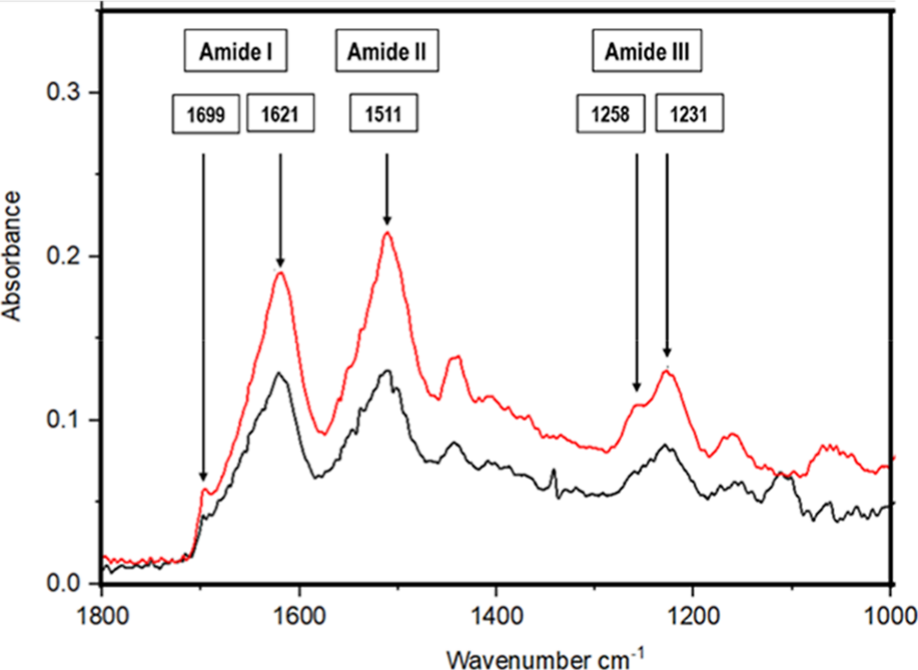
Expanded amide I, II, and III regions of the FTIR spectra of water (bottom spectrum)- and alkali (top spectrum)-degummed SF fibers.

Expanded amide I, II, and III regions of the FTIR spectra of water- and alkali-degummed SF fibers are given in Figure 9.

Both spectra are almost identical with well-defined amide I, II, and III peaks, indicating no significant effect of degumming medium and/or conditions on the SF structure. The crystallinity of the degummed SF produced was also determined by comparing the absorbance (A) values of amide II peaks located at 1258 and 1231 cm^–1^ [(*A*_1258_/*A*_1231_) × 100] and found to be 56% and 58% for water- and alkali-degummed samples, respectively, similar to those reported in the literature.^51,52^

### Morphology of Electrospun SF/PLA Composite Fiber Webs and Water Contact Angles

**Figure 10:**
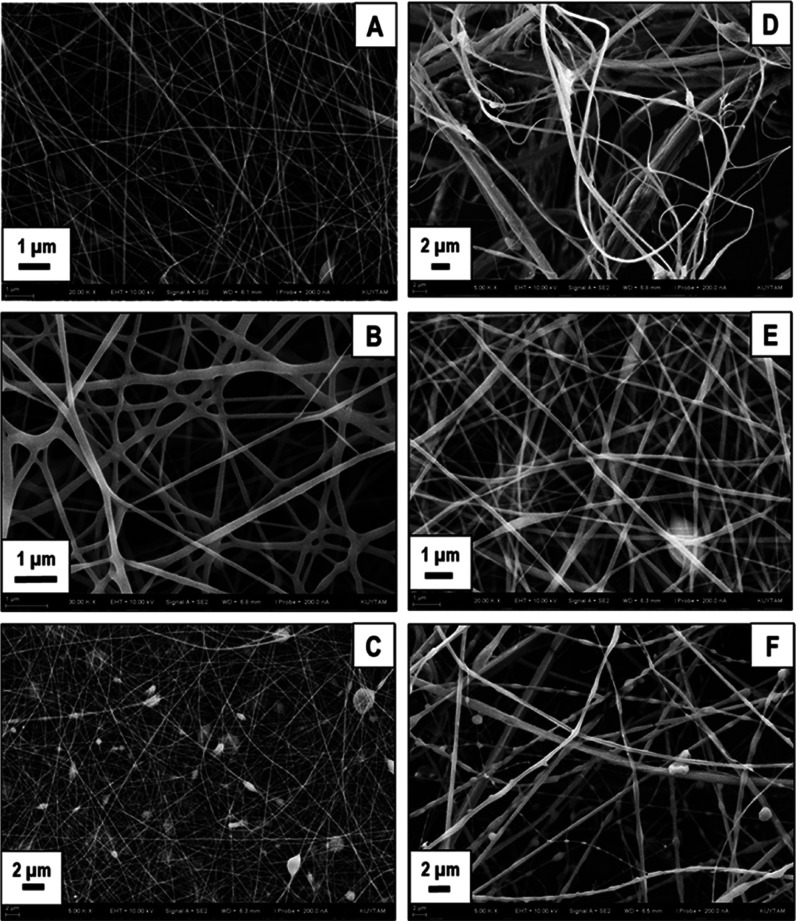
SEM images of electrospun SF/PLA blends dissolved in FA/OPA mixtures with different compositions. (A) W-7030, (B) W-8515, (C) W-9010, (D) A-7030, (E) A-8515, and (F) A-9010.

The SEM images of electrospun SF/PLA (7.5/1 w/w) webs prepared by using water (W)- and alkaline (A)-degummed SF and electrospun under identical conditions after dissolving in FA/OPA mixtures with different compositions are provided in Figure 10A–F.

As can be seen in Figure 10A–F, at all solvent compositions, it was possible to obtain electrospun webs, displaying fairly homogeneous fibrous structures. Fiber diameters obtained were generally in the 100–300 nm range. As the amount of OPA decreased in the mixture, bead formation is observed (Figure 10C,F) in both water- and alkaline-degummed SF. This can be attributed to the decreased solubility of SF as the amount of OPA decreases in the solvent mixture.

Static water contact angles obtained on electrospun SF/PLA webs are listed in Table 1. All electrospun webs show hydrophilic behavior with water contact angles in the 31–49° range regardless of the source of SF or FA/OPA solvent composition used in the electrospinning process. As the OPA ratio in the FA/OPA solvent mixture increases, the contact angle decreases in all SF/PLA electrospun scaffolds. A decrease is more prominent in water-degummed samples in comparison to alkali-degummed SF samples. This could be due to higher solubility of SF in OPA and its enrichment on the fiber surface during electrospinning or the presence of slightly higher amounts of residual OPA in SF.

**Table 1:** Static Water Contact Angles Obtained on Electrospun SF/PLA Webs

sample code	contact angle (deg)
W-9010	49
W-8515	31
W-7030	31
A-9010	48
A-8515	41
A-7030	38

### In Vitro Cell Viability Assay Results

**Figure 11:**
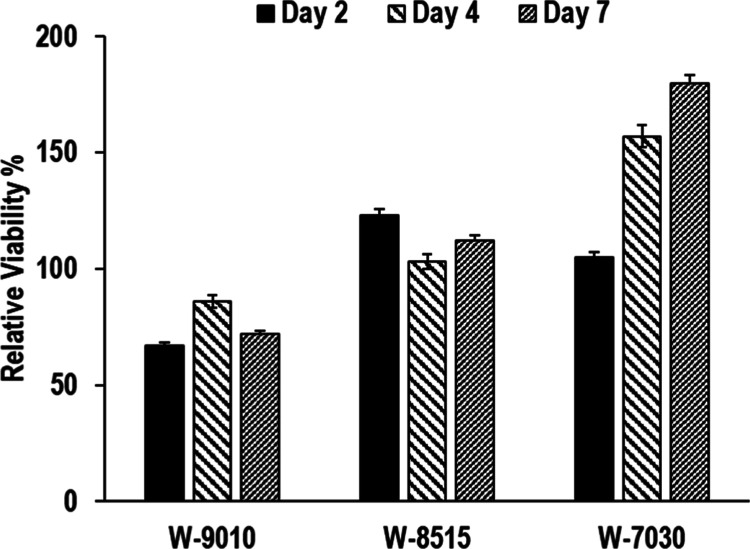
Relative in vitro cell viability results obtained on days 2, 4, and 7 on electrospun PLA/SF samples based on water-degummed SF.

In vitro cell viability assays were performed by seeding the HepG2 hepatocarcinoma cell line on electrospun SF/PLA biocomposite samples prepared by using water degumming and different FA/OPA solvent compositions. MTT reagent was applied on the seeded samples on days 2, 4, and 7, and UV absorbance readings were obtained. An empty cell culture plate without any samples was seeded with cells, and readings obtained in the same manner were taken as reference with 100% viability for days 2, 4, and 7. The results obtained are listed in Figure 11.

As can be seen in Figure 11, all samples perform well regarding the cell viability after 2, 4, and 7 days. All samples show viability values above 70–85% over 2 days, which increases to 100–125% after 4 days and up to 175% after 7 days for the W-7030 sample. The FA/OPA solvent composition does not seem to play a significant role in cell viability. These results clearly demonstrate that the water degumming method is a very effective process to produce bioactive SF/PLA scaffolds. The FA/OPA mixture used in SF dissolution, which replaces salts such as CaCl_2_ and LiBr used conventionally, may also result in higher cell adhesion and growth due to the bioactivity of phosphates.

## Conclusions

As a widely studied biomaterial for various biomedical applications, there is growing interest in developing alternative “green” processing techniques to produce SF. In this study, we demonstrate complete removal of the sericin layer on silk fibers in boiling water to generate SF, without any alkali treatment. SF generated is easily dissolved in a mixture of weak acids, FA/OPA (at 90/10, 85/15, and 70/30 v/v ratios) without using salts, such as LiCl and CaCl_2_, which need to be removed from the system by dialysis and freeze-drying. Water degumming and FA/OPA dissolution method has shortened the SF regeneration process to less than 24 h, when compared with alkali degumming and salt removal, which takes at least 3 days. Our results show that water degumming is a simple, effective, and green method for sericin removal. Furthermore, we have also demonstrated that well-defined fibrous SF/PLA scaffolds could be fabricated from FA/OPA solutions by electrospinning. Electrospun SF/PLA scaffolds exhibit high cell viability and cell growth. These results clearly show that an alkaline reagent and salt-free, simple, and green boiling water degumming method for sericin removal followed by dissolution in FA/OPA mixtures is a very effective production method for SF that can be used in tissue engineering applications.
